# The effects of high intensity interval training on appetite management in individuals with type 2 diabetes: influenced by participants weight

**DOI:** 10.1007/s40200-019-00396-0

**Published:** 2019-05-15

**Authors:** Saleh Afrasyabi, Syed Mohamad Marandi, Mehdi Kargarfard

**Affiliations:** Department of Exercise Physiology, Faculty of Sport Sciences, University of Isfahan, Hezar Jerib Street, P.O. Box 81746-7344, Isfahan, Iran

**Keywords:** Appetite peptides, Interval training, Obesity, Type 2 diabetes

## Abstract

**Background and purpose:**

The connection between exercise and appetite has ramifications for acute energy balance and weight-management. Research would suggest that exercise training can transiently suppress appetite, particularly in overweight and T2D, healthy-weight individuals. However, the effect of such a transient appetite suppression on subsequent food intake may be restricted. The aim of this thesis was to investigate appetite responses to HIIT in obesity with T2D and to assess the effect of other exercise characteristics, as well as exercise intensity, in mediating these responses especially appetite hormones.

**Materials and methods:**

Eighty individuals with type 2 diabetes (forty normal and forty obesity weight) performed HIIT trials, all in arandomly divided, in 8 groups (10 in each group) which included, obesity non-diabetic control, obesity diabetic control, normal weight diabetic control, obesity non-diabetic training, obesity diabetic training, normal weight, non-diabetic training, and normal weight diabetic training. Twelve-weeks HIIT sessions (each session of an interval training includes 60 s of high intensity training (85–95% of reserve heart rate)) + running for 60 s at low intensity (55–60% of reserve heart rate) were applied. Blood samples were taken at the beginning and after the fourth, eighth and twelfth week of the training. Data were analyzed using repeated variance analysis and Pearson correlation coefficient.

**Results:**

The results showed that training reduced ghrelin plasma levels in obese diabetic subjects (*P* < 0.05). Training has reduced PYY plasma in healthy subjects (non-diabetic) with normal weight (P < 0.05). Training reduced plasma levels of PYY in diabetic patients with normal weight and increased it in obese diabetic and healthy subjects (*P* < 0.05). Training has increased GLP-1 plasma in obese diabetic and diabetic with normal weight groups (*P* < 0.05). Training reduced TNF-α in normal (non-diabetic) subjects with normal weight and diabetic and non-diabetic obese subjects.

**Conclusion:**

Collectively, the studies reported here suggest that appetite hormones differ between lean and obesity participants. The finding also suggested HIIT is more likely to elicit appetite hormones responses in obesity than in lean individuals with type 2 diabetes. Therefore, with caution, it is recommended that the high intensity interval training can be beneficial for these patients.

## Introduction

Type 2 diabetes mellitus (T2DM) is an extended metabolic disease recognized by hyperglycemia and, triggered by insulin resistance and diminished insulin release. Medical management of T2DM is composed of nutrition treatment, medicinal therapy, and exercise. In obesity and type 2 diabetes, changed responses of these hormones happen. For example, in everyone with type 2 diabetes, fasting plasma ghrelin levels are typically reduced and decrease less in reactions to a meal [1–3]. Fasting and postprandial PP and PYY levels are lower in obese individuals [4, 5], and individuals with type 2 diabetes have been displayed to have diminished postprandial fullness [3]. These undesirable modifications in appetite and satiety control are not permanent, as a short-time session of aerobic exercise has been shown to enhance postprandial fullness in everyone with type 2 diabetes, with no changing acylated ghrelin levels [3]. Further, Recent evidence discovered that long-term exercise training improved PP concentrations [6] and intermittent exercise decreased hunger and enhanced satiety in obese non-diabetic participants [7]. High-intensity interval exercise training (HITT), which involves repeated bursts of strenuous exercise interwoven with intervals of recovery, may be an appealing option in applying a high-intensity exercise training strategy in T2DM. Body weight is managed by using the stability between energy consumption and energy expenditure. For weight manage, many researchers and scientists recommend regular exercise in order to enhance energy spending. Additionally, recent scientific studies demonstrate that exercise can modify energy intake with the adjustment of the energy-regulating hormones ultimately [1, 8–10].

Recent evidence suggests that Appetite control (hunger and satiety) is a complex physiologic process regulated by peptides secreted from the organs (stomach, pancreas, intestines, etc.) [11]. Eating can stimulate or suppress the secretion of several gastrointestinal hormones [12]. Stimulating hormones secretion is associated with digestive tract motility, gastric acid secretion from pancreatic enzymes, stimulation of gallbladder contraction and food intake.

Previous studies have reported that, Ghrelin, PYY, and GLP-1 are important hormones secreted from the gastrointestinal tract. Hunger is as a result of the ghrelin appetite peptide present in blood circulation in both acyl and non-acyl forms [13]. Acyl ghrelin affects appetite, while non-acyl ghrelin has no effect on appetite [13]. When hungry, the levels of ghrelin rise in blood circulation and it decrease after eating [14]. Satiation is caused by the hormone secreted from the pancreas PYY. During hunger, its plasma concentration decreases, while after eating, its concentration increases and then stimulates a sense of satiety ordering to stop eating [15, 16]. A number of researchers have reported that GLP-1 hormone is secreted from the small and large intestine and is released into the bloodstream after a meal, and its effects on the arteries of the hypothalamus apply through the GLP-1 receptor and the GLP-1 neurons with Single neuron transmitter (NST) and Vagus nerve [17, 18]. The effects of appetite regulation of this factor have been shown in humans [19, 20]. GLP-1 also causes delayed gastric emptying and reduced food intake and inhibits glucagon production [1]. Previous studies have indicated that changes in appetite peptides during diabetes and obesity are different. For example, the levels of ghrelin decrease in response to a meal in diabetics and obese people [8–10].

Moreover, GLP-1 levels are naturally low in T2D patients. PYY levels are also low in fasting and post-meal states in diabetic patients [7], indicating impaired satiety signals [10]. In addition to the effects of eating (satiety or hunger) on appetite peptides, previous studies have demonstrated that an increase in some inflammatory and hormonal factors affects the secretion of appetite regulating peptides [6]. For example, it was shown that higher IL-6 increases the production of GLP-1, secretion of intestinal L cells and pancreatic alpha [2, 6]. Therefore, we assumed that other inflammatory factors may also be effective on GLP-1 changes.

Recently, investigators have examined the effects of pro-inflammatory cytokine on inflammatory and autoimmune diseases. Among pro-inflammatory cytokine, TNF is a pro-inflammatory cytokine produced predominantly by monocytes and macrophages, playing an important role in inflammatory and autoimmune diseases. TNF expression in adipose tissues increases in obese and type 2 diabetes animal specimens, and has been confirmed in human specimens closely associated with type 2 diabetes, especially in obese subjects [3]. Released TNF involves the immune system in response to stimulus (obesity or type 2 diabetes) and changes brain activity. This cytokine changes the activity of the neural networks through a fast transmission pathway, causing drowsiness, fatigue, and loss of appetite [16–18].

Therefore, we looked at its relationship with GLP-1. On the other hand, we assumed that TNF-α may affect other endogenous and related gastrointestinal tract hormones. Thus, we examined the relationship between this inflammatory factor with ghrelin and PYY. Previous studies have shown that various physical activities can alter tissue gene expression and plasma concentration of appetite peptides [4, 5, 21, 22].

Although aerobic exercise is generally recommended for people with diabetes and obesity, it should be noted that variety of training in terms of form, duration and intensity may have different effects. High intensity interval training on the treadmill is an aerobic training which has not been studied much regarding its effects on diabetic and obese individuals. It may vary appetite peptides in people with type 2 diabetes. Therefore, the major objective of this study was to investigate the effect of a 12-week high intensity aerobic training on plasma concentrations of ghrelin, PYY and GLP-1 and its association with TNF-α inflammatory factor in diabetic and obese individuals.

## Materials and methods

### Subjects

Adults with T2DM were recruited by contacting local diabetes patient hospital. First, subjects were involved in the study and randomized inside eight groups: Obesity Non Diabetic Control (O-ND-C), Obesity Diabetic Control (O-D-C), Normal Weight Non Diabetic Control (N - ND - C), Normal Weight Diabetic Control (N - D - C), Obesity Non Diabetic Training (O - ND - T), Obesity Diabetic Training (O - D - T), Normal Weight Non Diabetic Training N - ND - T), and Normal Weight Diabetic Training (N - D - T). each group consist of 10 participants. Their mean age was 40 ± 10 years, mean height was 170 ± 10 cm, mean body mass index (BMI) was ≥30 kg·m − 2 for obesity and was ≤20 kg·m − 2 for lean, and mean duration of diagnosis was 3.0 ± 2.2 years. All volunteers were selected from university Hospital outpatients with result of a 2-h glucose tolerance test of 140 mg/dL ≤ blood sugar ≥200 mg/dL and no other complicating diseases. All volunteers underwent medical screening, including a health status interview, physical activity, smoking habits, alcohol consumption and diet, and blood analysis. Written informed consent was obtained from all subjects. Also, no subjects received insulin therapy, and no medications were altered during the exercise treatments.

Exclusion criteria were: moderate-high intensity exercise >1.5 h per week, diabetes duration <1 year, BMI < 30 kg/m2 for obesity and ≥ 20 kg/m2 for lean volunteers, use of exogenous insulin, evidence of renal, liver, cardiopulmonary, neuromuscular and/or psychological disease, other debilitating diseases or contraindicating physical activity. Moreever, there were two eligibility tests during the visits: 1) if markers or analytes as given below in detail did not fulfil criteria ranges, subjects were excluded and 2) if there were any perturbations during the heart cycle electrocardiogram (ECG) of both resting and working myocardium, subjects were excluded. All 80 T2D patients were under treatment with oral antidiabetic agents, either with metformin (*N* = 48) or glimepiride (*N* = 32). Additionally, lipid-reducing agents (*N* = 31), anti-hypertensive agents (*N* = 19), glucagon-like peptide-1 (GLP-1) receptor agent (*N* = 20) and glucagon-like peptide-1 (GLP-1) inhibitor agent (*N* = 10) were taken on a daily basis.

The present study was approved by the Faculty of Physical Education of the University of Isfahan and received written consent from the participants (CSEP). The present research also has the ethics code number IR.UI.REC.1396.058 from the Ethics Committee of the University of Isfahan.

### Study design

**Fig. 1:**
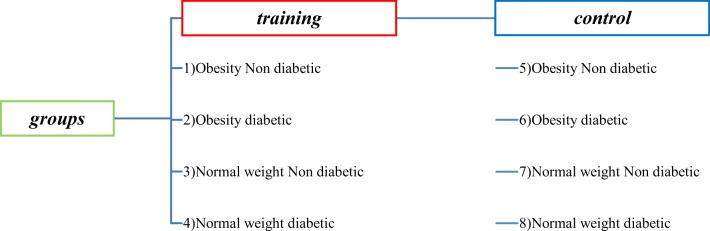
Grouping the participants in the study

**Fig. 2:**
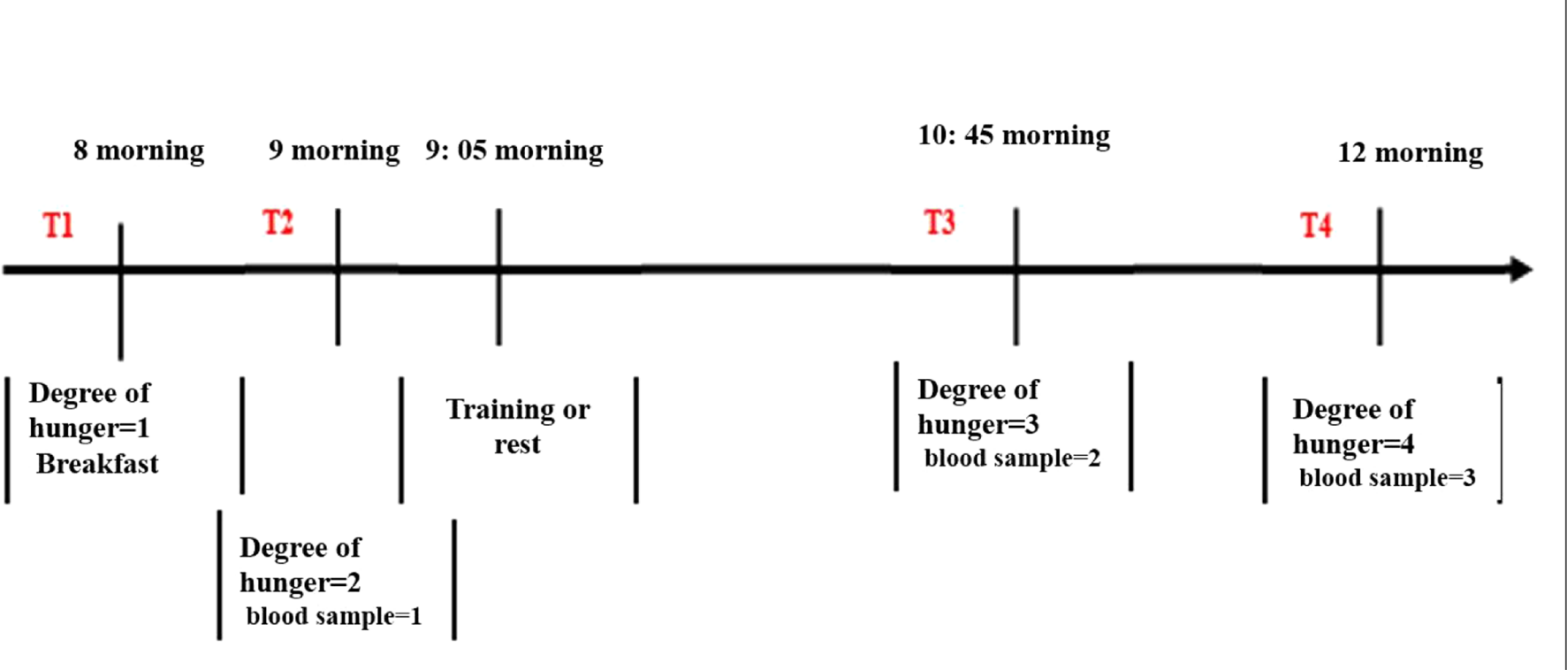
Timeline of twelve weekly sessions of interval activity. T1 Breakfast, T2 Pre-Tests, T3 Post Test, and T4 Recycling. 1. Hunger severity/breakfast. 2. Hunger severity/blood sampling. 3. Training or rest/control. 4. Hunger severity/blood sampling. 5. Hunger severity/blood sampling

The subjects referred to the laboratory for four consecutive sessions with a 2-day interval. Subjects were advised to refrain from any heavy exercise 48 h before the initial test. Anthropometric measurements (height, body weight, body composition, body mass index and fat percentage) and physiology of all subjects were evaluated in these four sessions in the laboratory. The percentage of subjects’ fat was measured using an electrical bio-impedance device (model 3/3, Olympia, South Korea). The peak oxygen consumption (VO2peak) of the subjects was measured on the treadmill by the bruce modify test. At the next sessions, subjects were assigned into eight groups (10 in each group) according to the criteria set out in the study (Fig. 1). As shown in Fig. 2, the event timeline is displayed for each session. At 24 h before the first session of the training program, participants received a meal with beverage representing the same diet on the day before the test. At 8 o’clock, the participants were fasting when arriving the laboratory (they did not receive any food or drink other than water for 10 h).

After determining the level of hunger, the participants consumed a standard 4-k calorie meal, 16.7 kJ per kg body weight. Breakfast included jam (250 kcal, 1046 kJ, 44 g carbohydrate, 9 g protein and 5 g fat) plus rice cake (35 kcal, 144 kJ, 7 g carbohydrate, 1 g protein and 0 g fat) and butter (200 kcal, 8/836 kJ, 7 g carbohydrate, 8 g protein and 15 g fat).

Then, the participants rested for 60 min for blood sampling, and their hunger level was measured based on the VAS index. Participants then began their training program based on the intensity and duration of the appointment. At the beginning of the session, the participants completed 5 min of warm up, and at the end of the session, they also performed 5 min of cool down, in addition to the intensity and duration of the training. After the end of the training session, a blood sample was immediately taken. A blood sample was also collected at an hour after the end of the activity during recovery.

### Hunger ranking

Hunger ranking was performed using VAS. Participants displayed the level of hunger using a vertical line along the 150 mm standard. The left-hand side of the domain indicated absence of hunger, and the right side indicated a high level of hunger [23]. Participants could drink water during the training session.

### Peak oxygen consumption assessment (vo2peak)

To evaluate peak oxygen consumption (vo2peak), all participants performed an incremental exhaustion test (Bruce Modified Protocol) on a programmable treadmill, while the gas analyzer was used. To demonstrate the achievement of vo2peak, the following conditions should occur: The peak oxygen consumption by increasing workload, the respiratory ratio rate greater than 1.15, the perceived pressure rate of greater than 17, and voluntary fatigue. The energy consumption of each session was about 300–400 kcal, measured by the consumption of vo2. All participants used the heart rate monitor during each training session to control the target heart rate.

### High intensity interval training program

Each high intensity training session consisted of 60 s of high-intensity running (95–85% of reserved heart rate) followed with 60 s at low intensity (55–60% of heart rate) during recovery. Participants performed six high intensity intervals during the first week, 8 intervals during the second week, 10 intervals during weeks 3 to 8, and 12 in the final 4 weeks. Five minutes of low intensity running for warm up at the start and 3 min of recovery was conducted at the end of each training session.

**Fig. 3:**
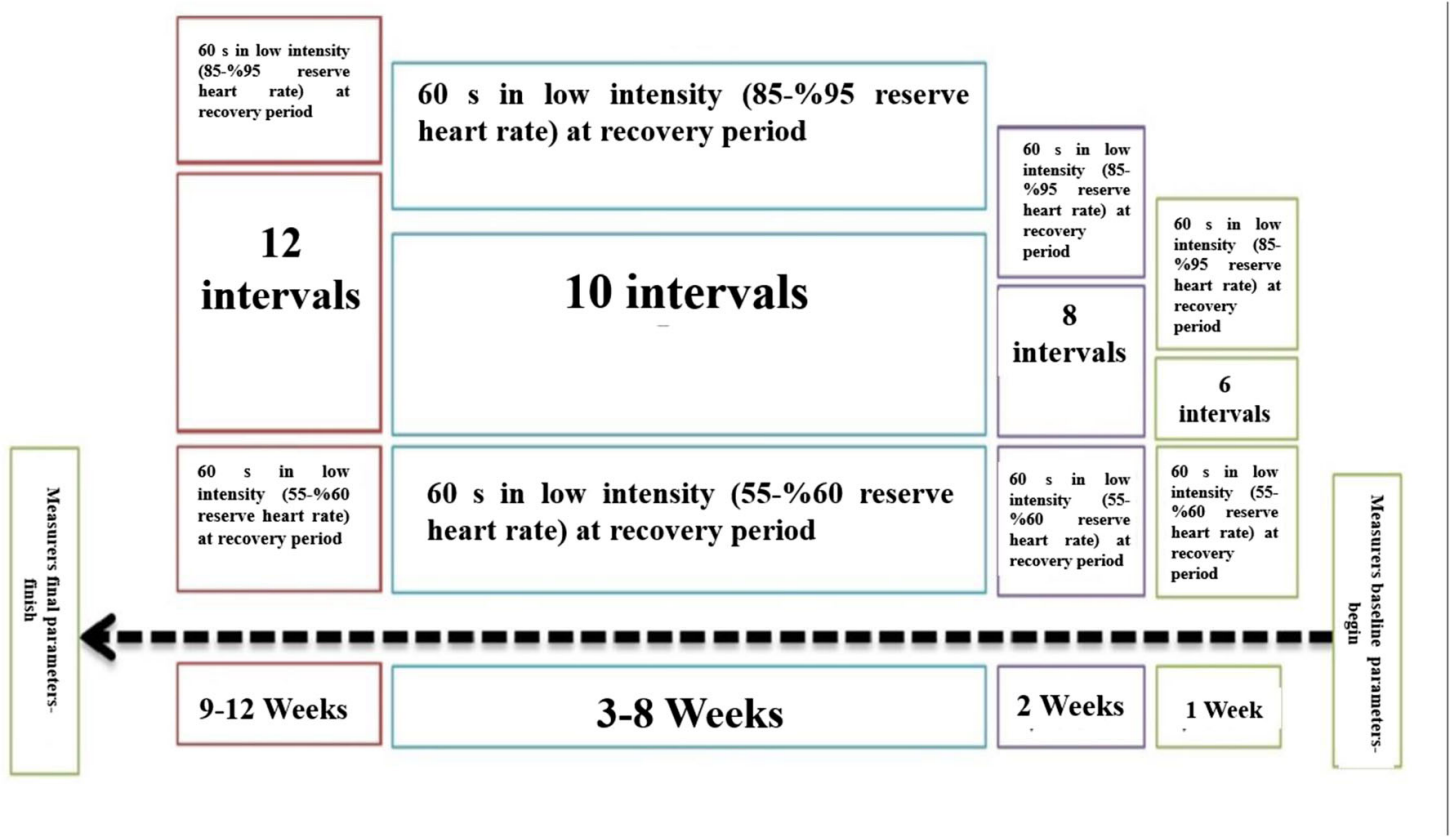
Twelve weeks intensive training on treadmill

Therefore, the original training lasted roughly between 20 and 24 min. The training program was conducted for 12 weeks, three sessions per week (Saturday, Monday, and Wednesday) under the supervision of a sports physiologist, as shown in Fig. 3.

### Biochemical analyses

All analytic processes were conducted according to the manufacturer’s instructions. Plasma concentrations were determined using the ELISA kit in accordance with the manufacturer’s kit. Plasma acyl ghrelin levels were measured by sandwich ELISA using the human kit of the German company Zellbio. The sensitivity of this method was 7.81 pg/ml, and the percent of intra-test variance was 8.1%. The ELISA GLP-1 and PYY kit from the company of the Republic of Sweden, with a sensitivity of 1 pg/ml per liter and 20 pg / ml were used for intra-group variation coefficient which were less than 10 and 5%, respectively. The IL-6 and TNF-α ELISA kits of the Daikon Company of France, with a sensitivity of 0.3 and 8 pg / ml and the intra-group variation coefficient of less than 9 and 6%, respectively, were used.

### Calculations and statistical analyses

Data were analyzed using descriptive statistics as mean ± standard deviation. The Kolmogorov-Smirnov test was used to determine natural distribution of data, and repeated variance analysis test was used to compare intra-group and inter-group changes, and Pearson correlation coefficient was utilized to determine the relationship between the variables. Data analysis was performed using SPSS software version 19 and all significant levels were considered as *P* < 0.05.

## Results

Eighty lean and obese men with type 2 diabetes were recruited for this study (Table 1). Half of participants were lean and obese, physician diagnosed with type 2 diabetes, not using insulin, and undergoing standard medical care (Table 2).

**Table 1:** Characteristics of subjects

Variable	O-ND-T	O-D-T	N-ND-T	N-D-T	O-ND-C	O-D-C	N-ND-C	N-D-C
Age (year)	43.26 ± 6.88	44.57 ± 5.58	42.70 ± 5.02	43.51 ± 5.81	46.20 ± 6.87	41.23 ± 4.06	44.33 ± 5.26	47.02 ± 5.58
Height (cm)	175.13 ± 7.63	174.31 ± 7.26	175.76 ± 5.65	174.35 ± 5.94	171.84 ± 5.02	177.31 ± 4.11	176.02 ± 5.93	176.42 ± 2.68
Weight (kg)	104.65 ± 2.24	100.23 ± 4.21	64.19 ± 5.35	63.03 ± 5.76	98.48 ± 3.15	101.70 ± 3.48	60.71 ± 4.24	64.69 ± 9.69
BMI (kg/m2)	33.88 ± 2.61	33.08 ± 2.40	20.82 ± 2.10	20.74 ± 1.73	33.44 ± 2.50	32.40 ± 2.02	20.30 ± 2.33	20.58 ± 3.22
fasting glucose (mmol/dl)	6.43 ± 0.80	6.26 ± 1.03	4.04 ± 0.62	5.15 ± 0.69	5.71 ± 0.56	5.99 ± 0.84	4.07 ± 0.67	4.29 ± 0.97

**Table 2:** Correlation between variables

Variable	Ghrelin (b)	Ghrelin (4)	Ghrelin (8)	Ghrelin (12)	PYY (b)	PYY (4)	PYY (8)	PYY (12)	GLP-1 (b)	GLP-1 (4)	GLP-1 (8)	GLP-1 (12)	TNF-α (b)	TNF-α (4)	TNF-α (8)
Ghrelin (4)	.722 **														
Ghrelin (8)	.744 **	.817 **													
Ghrelin (12)	.751 **	.832 **	.848 **												
PYY (b)	.112	−.193	−.076	−.115											
PYY (4)	.174	−.225 *	−.160	−.182	.763**										
PYY (8)	.183	−.219	−.101	−.148	.788**	.919 **									
PYY (12)	.277 *	−.176	−.122	−.122	.685**	.912 **	.897 **								
GLP-1 (b)	.448 **	.415 **	.338 **	.423 **	−.160	−.099	−.148	−.053							
GLP-1 (4)	.495 **	.312 **	.242 *	.278 *	−.080	.041	−.018	.141	.610 **						
GLP-1 (8)	.543 **	.494 **	.359 **	.352 **	−.116	−.026	−.091	.069	.574 **	.644 **					
GLP-1 (12)	.581 **	.340 **	.341 **	.261 *	.064	.148	.128	.262 *	.543 **	.655 **	.652 **				
TNF-α (b)	−.055	−.252 *	−.099	−.165	.257*	.231 *	.267 *	.170	−.018	.057	−.074	.072			
TNF-α (4)	−.195	−.368 **	−.266 *	−.212	.244*	.241 *	.219	.113	−.129	−.192	−.248 *	−.168	.584 **		
TNF-α (8)	−.163	−.207	−.152	−.122	.211	.077	.122	.001	−.179	−.252 *	−.328 **	−.238 *	.616 **	.720 **	
TNF-α (12)	.039	.089	.086	.211	−.214	−.263 *	−.326 **	−.283 *	.026	−.122	−.170	−.229 *	.274 *	.492 **	.571 **

**. Correlation is significant at the 0.01 level. *. Correlation is significant at the 0.05 level

**Fig. 4:**
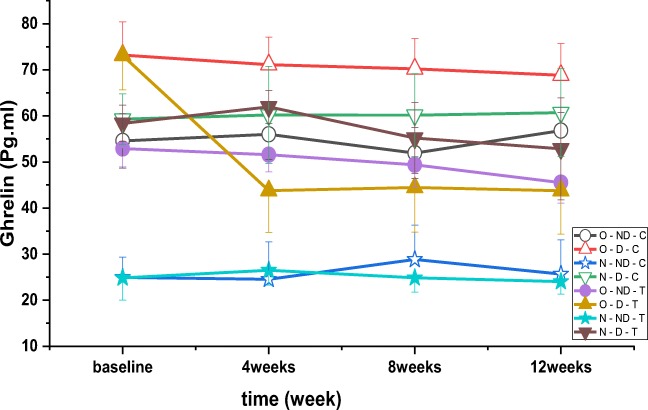
Variations in ghrelin levels (pg.ml-1) after 4, 8 and 12 weeks of high intensity interval training

There were significant differences between ghrelin over time (*P* = 0.002, F = 10.791), between the groups (*P* = 0.001, F = 179.489) (Fig. 4) and interaction groups*time (*P* = 0.034, F = 2.328). The results of post hoc test indicated that plasma ghrelin concentration in obese diabetic (*P* = 0.001), diabetic (*P* = 0.001), normal weight diabetes with training (*P* = 0.001), obese diabetes control (*P* = 0.001) diabetic (*P* = 0.001), and normal control with diabetes (*P* = 0.001) was higher than normal control without diabetes. The results of post hoc test showed that ghrelin concentration was significantly lower in obese diabetic (*P* = 0.001), diabetic (*P* = 0.001) and normal weight no-diabetic (*P* = 0.001) training groups compared to normal weight diabetic control. Also, ghrelin concentration was significantly higher in obese diabetic control group compared to normal weight diabetic control (*P* = 0.001). Post hoc test showed that plasma concentration of ghrelin in obese non-diabetic group (*P* = 0.001), diabetic (*P* = 0.001), obese control (*P* = 0.001), normal weight without diabetes (*P* = 0.001), and normal weight diabetics control was significantly lower than control obese diabetics (*P* = 0.001).

**Fig. 5:**
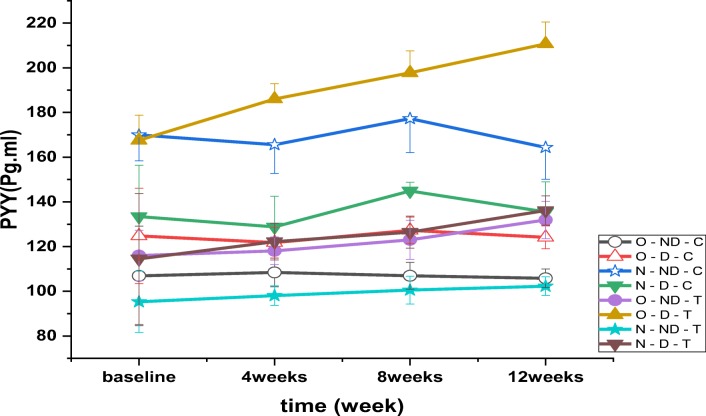
Changes in plasma PYY levels (pmol.l-1) following 4, 8 and 12 weeks of high intensity interval training

The results revealed a significant between PYY in time (*P* = 0.001, F = 14.004), between the groups (*P* = 0.001, F = 254.075) (Fig. 5) and interaction groups*time (*P* = 0.001, F = 3.438). Post-test results indicated that plasma PYY concentration was lower in all groups except for obese diabetic training than control normal without diabetes (*P* = 0.001). The results of post hoc test showed that PYY concentration in O-ND-T (*P* = 0.001), N-ND-T (P = 0.001), N-D-T (*P* = 0.004), O-ND-C = 0.001) and O - D - C (*P* = 0.001) was significantly lower than N - D - C. Also, PYY concentration in the O - D - T (*P* = 0.001) and N - ND - C (P = 0.001) groups was higher than N - D - C. The results of post hoc test showed that PYY concentration in the O-D-T (*P* = 0.001), N-D-T (P = 0.001), N-ND-C (P = 0.001) and N-D-C = 0.003) was more than O-D-C and in O-ND-C group (*P* = 0.001) was lower than O-D-C. Post hoc test results indicated that PYY concentration in all groups was significantly higher than O - ND - C except N - ND - T (*P* = 0.001).

**Fig. 6:**
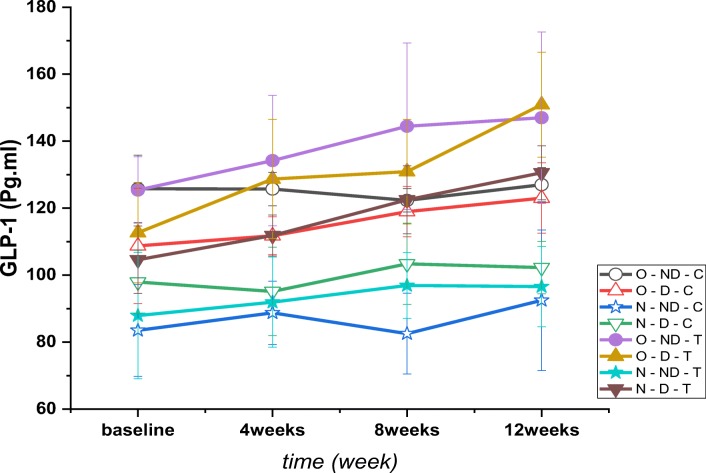
Changes in plasma GLP-1 levels (pg.ml-1) following 4, 8 and 12 weeks of high intensity interval training

The results revealed a significant between GLP-1 in time (*P* = 0.001, F = 19.195), between the groups (*P* = 0.001, F = 67.249) and interaction groups*time (P = 0.001, F = 4.204) (Fig. 6). Post hoc test results indicated that plasma GLP-1 concentrations were higher than N-ND-C in all groups except N-ND-T (*P* = 0.001). Post hoc test results showed that GLP-1 concentrations were higher than N-D-C in all groups except N-ND-T and N-N-D (*P* = 0.001). Also, the concentrations of GLP-1 in N-ND-T (*P* = 0.001) and N-ND-C (*P* = 0.001) groups were lower than N-D-C. The results also showed that the concentrations of GLP-1 in O-D-T (*P* = 0.001) and O-D-T (*P* = 0.001) groups was higher than O-D-C and in the N-ND-T (*P* = 0.001) and N-ND-C groups were lower than O-D-C. Post hoc test results showed that the GL-1 concentrations were significantly lower than O-ND-C in all groups except O-ND-T, O-D-T, N-D-T and O-D-C. (*P* = 0.001).

**Fig. 7:**
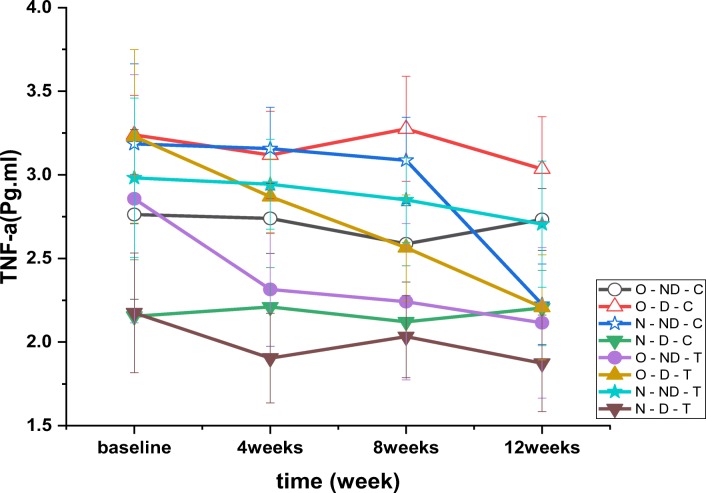
Plasma TNF-α levels variations (pg.ml-1) after 4, 8 and 12 weeks of high intensity interval training. Results of correlation between variables are presented in Table 2

The results of TNF-α test showed a significant effect over time (*P* = 0.001, F = 25.588). Moreover, the results for TNF-α showed a significant difference between the groups (*P* = 0.001, F = 34.427) (Fig. 7). It was found that changes in groups over time were also significant (*P* = 0.001, F = 4.279). The results of post hoc test indicated that TNF-α plasma levels were lower than N-ND-C except for O-ND-T, N-D-T and N-D-C groups (*P* = 0.001). Post hoc test results showed that TNF-α concentration was higher in all groups than N-D-C except for O-ND-T and N-D-C (*P* = 0.001). The results of post hoc test revealed that TNF-α concentrations was lower in O-ND-T (*P* = 0.001), O-D-T (*P* = 0.001), O-ND-C (*P* = 0.001) and N-D-C (*P* = 0.001) than O-D-C. The results of post hoc test showed that TNF-α concentrations was significantly lower in O-ND-T (*P* = 0.025), N-D-T (*P* = 0.001), N-D-C (*P* = 0.001) than O-ND-C (*P* = 0.001) and it was significantly higher in N-ND-C (−0.71), O-D-T (*P* = 0.001), N-ND-T (*P* = 0.001) O-D-C (*P* = 0.001) than the O-ND-C group.

## Discussion

Most importantly, in this non-invasive research, we have demonstrated that 12 weeks of low volume HIIT improves appetite control as determined by reduced average TNF-α, PYY and ghrelin concentration, and increased mean GLP-1 in T2D patients. This study delivered several important findings: Training has reduced ghrelin plasma levels in obese diabetic individuals. Training has reduced PYY plasma in healthy subjects (non-diabetic) with normal weight. Training has reduced PYY plasma levels in diabetic patients with normal weight. Training has increased plasma PYY in obese diabetic and obese healthy individuals. Training has increased GLP-1 plasma in obese diabetic and diabetics with normal weight. Training has reduced TNF-α in healthy subjects (non-diabetic) with normal weight. 6- Training has reduced TNF-α in obese diabetic and obese non-diabetic subjects.

Although fewer exercise training scientific studies with appetite-related results have been conducted, these investigations are important to determine the efficiency of exercise as a lifestyle strategy for weight control. However, evidence investigating the chronic effects of aerobic exercise on appetite variables is largely contradictory. The ACSM and ADA advocate moderate- and strenuous-intensity exercise for individuals with T2DM and supply evidence-based recommendations for prescribing exercise training, in terms of intensity, volume, frequency, duration, and rate of development. In this research, the variable of intensity was same between the groups. On the basis of the conclusions of this research, people who have type 2 diabetes and engage in exercise training at low volume HIIT, as characterized by the ACSM and ADA, may experience similar gains in appetite hormones [16–18].

Inactive diabetic patients document numerous limitations to physical activity, however the most typical explanation given is a “lack of time” for exercise. Nevertheless, there is an increasing body of literature that analyzes the metabolic risk factor lowering effects of traditional endurance training programs with the decrease of risk caused by unique exercise treatments that demand minimal amounts of time for patients. For this reason, HIE was distinguished as a successful damage that increases on the tediousness of low-intensity exercise training [18, 19].

In spite of contradictory findings, it has been recommended that chronic exercise adjusts the sensitivity of the appetite control system by balancing the increased drive to eat with an improved satiety response to a meal. A small collection of studies has shown an increase in concentrations of acylated ghrelin, PYY, GLP-1, and PP. duty of GLP-1 in the therapy of obesity and T2DM has appeared. GLP-1, an incretin hormone which controls blood glucose amounts, boosts insulin sensitivity by stimulating the pancreas to secrete insulin and is reported to increase the hydrolysis of triglyceride in adipose tissue. GLP-1 seems to be destroyed in T2DM patients [1]. Additionally, earlier exercise-related researches have revealed that healthy people have increased levels of incretin hormones such as GLP-1. Individuals who are obese displayed similar results to people with normal weight through weight loss resulting from exercise [9]. As exercise is regarded to be the initially line of interference for the avoidance and control of T2DM, enhancing GLP-1 through exercise is of the maximum importance. GLP-1, released through food stimulation, is decayed at a very high speed by the enzyme dipeptidyl peptidase-4 (DPP-4), and its involvement in the physiological regulation of eating causes weight loss. In this study, diabetes groups (obesity and lean individuals) were found to have significantly increased levels of GLP-1. Additionally, the HIIT groups appearing to have a greater increase in GLP-1 than control groups.

In the present investigation HIIT alter subjective ratings of appetite in after exercise program. That is, HIIT stimulate any latent changes in appetite. Some recent work has been published suggesting that appetite may be stimulated by exercise in the later hours of recovery. Consistent with no change in appetite, the present study found significant effects of HIIT on energy intake. When the present study was conducted there were few other data available regarding the effects of HIIT on energy intake although one recently published study has since provided some preliminary data. Previous studies have shown that ghrelin is a 28-amino acid peptide, more than 70% of which is secreted from gastric fundus cells [24]. In addition, the stomach, pancreas, kidneys, pituitary and intestines can also secrete this hormone [24, 25]. In general, training causes blood to go to the skin and reduces the blood flow to the viscera. Therefore, ghrelin loss during activity can be due to a reduction in the blood flow in the stomach and intestine as they are involved in acylation of ghrelin [26]. Ghrelin is secreted by the peripheral tissues and can penetrate the central nervous system (CNS). Therefore, it may have some central effects and affect different signaling paths. It has been shown that ghrelin exerts its central activity through the Vagus nerve. Ghrelin receptors have been seen at terminals of the Vagus. Increasing the Vagus nerve activity decreases the secretion of ghrelin into the blood circulation. It is possible that Vagus cell activity increases due to high intensity physical activity and it can reduce secretion of ghrelin from the stomach [27].

Surveys such as that conducted by Fathi et al. showed the effect of three different training intensities (low, moderate, and high) on ghrelin concentration in the gastric fundus tissue, skeletal muscle and plasma in adult male rats. They concluded that ghrelin was reduced in the fundus of the stomach by increasing the intensity of training in training groups compared to the controls [28]. The present study indicated that high intensity interval training on treadmill reduced ghrelin plasma in obese diabetic subjects. This is important as it indicates that high intensity interval aerobic activity can reduce obesity in obese diabetic subjects probably due to ghrelin suppression (since it has been reported that ghrelin increases body fat even independently of food intake [29].

Overall, previous studies showed that moderate intensity long-term training [30–32] and sprint interval training have increased PYY [33, 34] in men. The effects of exercise on PYY and GLP-1 have not been clearly identified. However, there are possible mechanisms. Increasing the sympathetic nervous system activity and catecholamines can affect PYY and GLP-1 and increase the secretion of these two hormones from the intestinal L cells [35, 36]. Another mechanism is the interleukin 6 effect. Previous studies have shown that IL-6 has increased GLP-1 in animals [37, 38]. The present study showed that there was a relationship between TNF-α levels and appetite peptides of ghrelin, PYY and GLP-1, and reinforced the idea that inflammatory factors can be considered as a mechanism for altering appetite peptides after training. The present study showed a significant negative relationship between TNF-α levels with ghrelin, PYY and GLP-1 in the 12th week. Of course, the relationship should not be discussed as a cause. To clarify this, there is a need for studies that specifically examine the effect of this inflammatory factor on appetite peptides.

This is well demonstrated recently that individuals with overweightness and obesity who lost more weight after a long-term exercise intervention displayed an elevated postprandial rise in GLP-1 and total PYY, and a greater suppression in acylated ghrelin compared to those who lost less weight. In present study obesity individual showed that more less weight than lean subjects which might result in more change in obesity with T2D [32–35]. Reasons for these discrepancies are unclear, and further studies are needed to clarify the variations in appetite-related hormones after exercise training that could be modulated by the loss body fat and weight of participants.

Previous studies showed that TNF-α gene is expressed both in white adipose tissue in animals and obese humans. TNF-α has a positive and strong correlation with body mass index and insulin sensitivity [39]. The literature revealed that TNF-α has decreased in the adipose tissue of obese mice by running [40]. However, some studies have shown an increase [41] and some a decrease of TNF-α [42] after training. Studies of obese people have shown that TNF-α in adipose tissue of obese subjects reduced after 15 weeks of training. However, some studies also showed that TNF-α had no significant change in obese patients after 12 weeks of aerobic training [43]. There are conflicting results on TNF-α blood levels [44, 45]. A study on diabetic subjects showed that 4 weeks of walking did not affect the normal weight diabetics on TNF-α, but reduced its concentration in obese diabetic patients [46]. Another study on obese women practicing on bicycles showed that 12 weeks of training reduced TNF-α in both insulin-resistant and non-insulin-resistant subjects [47]. Another study demonstrated that 12 weeks of training and dietary control had no effect on the levels of TNF-α in obese individuals [43]. In another study, 12 weeks of training led to an increase in TNF-α in female subjects [48]. Overall, it is understood that training has more effects on obese people TNF-α and the type of training is likely to be decisive. An important result of the present study is that the 12-week aerobic training program has been shown to reduce TNF-α in diabetic and obese with non-diabetic subjects. In conclusion, we showed positive and reliable results on the effect of an interval aerobic training on the regulation of ghrelin, PYY, GLP-1 and TNF-α. The relationship of appetite peptides with the TNF-α inflammatory factor should be considered as a possible mechanism that would require further study in the future.

## Conclusion

In conclusion, this study has shown that a chronic HIIT does influence appetite after 12 weeks HIIT program. Furthermore, HIIT alter energy intake at HIIT than control groups. HIIT stimulated a reduce TNF-α, PYY and ghrelin, also enhance GLP-1 hormones, however further research is needed to confirm this and to identify the responsible mechanisms. This data adds to knowledge regarding the specific influence of HIIT on energy homeostasis and appetite hormones.

## Abbreviations

O-ND-C: Obesity Non-Diabetic Control
O-D-C: Obesity diabetic Control
N-ND-C: Normal weight Non-Diabetic Control
N-D-C: Normal weight diabetic Control
O-ND-T: Obesity Non-Diabetic Training
O-D-T: Obesity diabetic Training
N-ND-T: Normal weight Non diabetic Training
N-D-T: Normal weight diabetic Training
